# Patients’ Perception of a Brief Web- and Mindfulness-Based Intervention for Pain Following Discharge After Total Joint Arthroplasty: Qualitative Description

**DOI:** 10.2196/69010

**Published:** 2025-08-06

**Authors:** Geraldine Martorella, Adam Wesley Hanley, Kathryn Elizabeth Muessig

**Affiliations:** 1 College of Nursing, Florida State University, 98 Varsity Way, Tallahassee, FL, 32306, United States, 1 8506459758, 1 8506447660; 2 College of Nursing, Institute on Digital Health and Innovation, Florida State University, Tallahassee, FL, United States

**Keywords:** pain, mindfulness, online, surgery, web, arthroplasty, patient perception

## Abstract

**Background:**

Important levels of pain are reported upon discharge from major surgery, with a risk of becoming chronic. Further, individuals express the need for support in managing pain after discharge. However, very few studies address pain management interventions in the postdischarge phase after surgery, including for individuals undergoing total joint arthroplasty (TJA). We have conducted a pilot randomized controlled trial testing a brief mindfulness intervention targeting people at risk for chronic postsurgical pain 2 weeks after surgery. Although the intervention we proposed was judged acceptable based on ratings obtained through a questionnaire, the nuanced perceptions of why and how it is considered acceptable are critical in refining the intervention. Moreover, the acceptability of mindfulness interventions in the perioperative context remains generally unknown and even more so in the postdischarge setting.

**Objective:**

The purpose of this study was to use qualitative data to explore the individual perception of acceptability of a brief 4-week, Web- and mindfulness-based intervention for pain following discharge after a TJA.

**Methods:**

A qualitative description was used to assess patients’ perception of the preliminary version of the intervention for pain management following discharge after surgery. The qualitative assessment was done at the end of the 4-week intervention (6 weeks after surgery). Semistructured interviews with open-ended questions were used to encourage free expression from participants (n=16) before proceeding to content analysis.

**Results:**

When reflecting on the benefits of the intervention, the main themes that emerged were mindfulness, pain acceptance, and supplementary relief. Overall, the intervention was perceived as relevant and suitable during recovery, although participants experienced a few challenges related to the novelty of mindfulness practice. Engagement and readiness were discussed in relation to adherence to the intervention. Addressing expectations and personal beliefs before the intervention could improve participants’ adherence. Offering additional support when spikes of pain occur could help overcome some challenges related to mindfulness practice during postoperative recovery.

**Conclusions:**

Given the increasing number of TJA surgeries performed annually and the effectiveness of nonpharmacological interventions, such as mindfulness-based approaches, in supporting recovery and well-being, efforts should be made to increase patient access to these promising adjunctive treatments. Combining nonpharmacological interventions before and after surgery may be an interesting avenue to optimize pain relief and recovery, as well as prevent complications. Finally, the use of technology could improve the accessibility, scalability, and adoption of these promising approaches for individuals with limited resources and mobility.

## Introduction

Pain after total joint arthroplasty (TJA) is expected, but little is known about pain after discharge [1]. As the length of hospital stay after surgery continues to decrease, it is essential to develop our knowledge regarding pain and its management in the subacute postoperative phase. A recent meta-analysis found the prevalence of moderate to severe postoperative pain ranges from 51%, one day after discharge, to 58%, 1‐2 weeks after discharge [1]. Two weeks after surgery marks the beginning of the subacute phase and is the time when pain and analgesic consumption are expected to decrease or when persistent postsurgical pain begins to be evident [2]. Hence, these pain rates may be unnecessarily high and put patients at elevated risk for developing chronic postsurgical pain (CPSP) [3,4], prolonged opioid use, and adverse effects of nonopioid agents [5-7]. Very few studies address pain management interventions in this setting, including after TJA [2,8,9], despite documented concerns and lack of support reported by individuals during their recovery following TJA [10]. Multiple barriers are also involved with this gap in the continuum of care [11,12]. Based on prior research with both clinicians and patients [12,13], there are promising opportunities for training postoperative patients in self-management of pain. Given the risks associated with pharmacological approaches, efficient nonpharmacological interventions for pain should be developed and offered to these individuals.

A variety of educational and psychoeducational interventions exist for individuals undergoing TJA that have been shown to have minimal impact on pain in the perioperative setting [9]. More recently, traditional 8-week mindfulness-based interventions (MBIs) have been delivered preoperatively to patients with TJA, and early evidence indicates they improve outcomes [14,15]. However, in addition to a lack of interest to engage in such interventions in the preoperative period, the accessibility and scalability of 8-week MBIs are limited by feasibility and acceptability challenges, including the substantial time commitment required to participate in an 8-week intervention, the extended gap between starting the intervention and undergoing surgery, and logistical barriers such as scheduling multiple treatment sessions over several weeks [14,15]. To address some of these limitations, our team and others have recently adapted MBIs to address the needs of patients in the preoperative period [14-23]. Brief MBIs delivered preoperatively have been shown to have medium to large effects on orthopedic surgical patients’ acute pain, pain unpleasantness, opioid use, and pain medication desire [19,20]. These novel brief MBIs are promising but have not been delivered in a targeted manner in the first few weeks after surgery to patients with a different postoperative pain trajectory associated with worse outcomes, showing warning signs for transitioning to CPSP [24].

However, providing patients with effective pain management in the subacute period after TJA is complicated by shortened postoperative hospital stays, mobility challenges, and uncertainty about who is responsible for pain management after discharge [11,25]. As a result, patients are often left to self-manage pain, which can be overwhelming and lead to inconsistent or inadequate care. In an effort to address barriers to the implementation of pain management interventions after discharge and to increase their accessibility, we proposed a Web-based asynchronous version of a brief MBI, which showed positive outcomes [26]. However, the acceptability of these MBIs, including individual experiences and perceptions in the perioperative context, remains generally unknown, and the very few studies address traditional 8-week formats [14,27]. Although the intervention we proposed was judged as acceptable based on ratings obtained through a questionnaire [26], the nuanced perceptions of why and how it is considered acceptable are missing. It is critical to capture this aspect to understand which intervention elements are perceived as effective and appropriate and how we can tailor the intervention and further improve its acceptability and efficacy. Therefore, the purpose of this study was to use qualitative data to explore the individual perception of acceptability of a brief 4-week MBI for pain following discharge after a major orthopedic surgery, for example, total hip or knee arthroplasty. The data and results presented in this paper are secondary to a pilot randomized controlled trial (RCT) that was conducted to evaluate the feasibility and preliminary effects of this intervention after TJA in 36 older adults [26].

## Methods

### Design

This study is part of a pilot RCT that examined the impact of a brief 4-week MBI intervention on pain and other postoperative outcomes [26]. Data collection was completed in August 2022. A qualitative description [28] was used to assess patients’ perception of the preliminary version of the intervention for pain management following discharge after surgery. This approach matched the needs of this study by allowing the analysis to stay close to the data and the informants’ point of view without imposing a specific philosophical or theoretical framework (eg, grounded theory and phenomenology) [28,29]. Qualitative descriptive analyses are particularly useful when developing and refining clinical interventions in the context of health services research, where patients’ perspectives are the ultimate evaluation [29-32]. This design allowed us to describe the extent to which the intervention is acceptable and what ingredients are helpful or need improvement from a patient’s perspective.

### Ethical Considerations

The project received ethics approval from Florida State University’s institutional review board in January 2022 (#STUDY00001771) and was preregistered (NCT04848428). Informed and written consent was obtained from each participant. During the initial meeting with the research team, besides providing details on the study and the nature of participation, it was emphasized that participating was completely voluntary and that participants could refuse or withdraw at any time without affecting their care. Strict measures were implemented in collaboration with our clinical partners to protect privacy throughout the study. Confidentiality and anonymity were also ensured by assigning a code to each participant during data collection, analysis, and the use of illustrative verbatim in the current manuscript.

### Procedure

Nurses from the preoperative clinic introduced the study to potential participants. If interested, an experienced research assistant (RA) explained the study, answered any patient questions, and signed the consent form during a preoperative visit 1 week before surgery. Two weeks after surgery (T0), the RA called each participant to confirm their eligibility and collect baseline sociodemographic and clinical data. Two weeks correspond to the first follow-up with the surgeon, and waiting 2 weeks before beginning the interventions also allowed participants to recover from acute surgical pain and experience the challenges of managing persistent pain in their daily activities. Participant inclusion criteria included the following: (1) 18 years and older; (2) first elective TJA; (3) presence of pain during movement ≥4/10 [33] 2 weeks after surgery; (4) ability to understand and complete questionnaires in English; (5) ability to use an electronic device such as a smartphone and computer or tablet; and (6) completed the brief 4-week MBI. Patients were not eligible for the study if they were unable to consent because of physical or mental incapacity. Participants received gift cards of US $25 at each of the data collection time points (T0: 2 wk after surgery, T1: 6 wk after surgery). After a prescreening and a baseline assessment, participants were randomized to receive the brief 4-week MBI (experimental group) or a pain-coping educational intervention (active control group). A total of 18 participants were assigned to the experimental group receiving the new brief Web-based MBI. An assessment of the acceptability of the intervention was conducted at the end of the intervention using a questionnaire. Semistructured individual interviews (telephone) were then conducted by the RA with all intervention arm participants to explore their individual perceptions. The sample size was thus not based on data saturation, but on involvement in the experimental group.

### Intervention

In addition to usual care, participants received the brief MBI delivered remotely, asynchronously through prerecorded videos. Across the 4 weeks, participants received a weekly link via email or text message that allowed them to access their intervention videos. The intervention videos were embedded in Qualtrics to allow optimized viewing from any type of device, for example, a computer, tablet, or smartphone. Participants were invited to watch the videos as much as needed or desired. Over the course of the 4-week intervention, participants received reminders to view their weekly video if they had not viewed it. In an effort to promote self-management, the intervention was self-guided, and participants received no other interventional support.

The brief MBI is an adaptation of mindfulness-oriented recovery enhancement (MORE) [34]. The traditional MORE program is an efficacious, 8-week treatment for chronic pain and opioid misuse [35-37] that integrates 3 core therapeutic elements (ie, mindfulness, positive reappraisal, and savoring) to promote positive patient outcomes. The brief, 2-hour version of MORE (ie, brief mindfulness-oriented recovery enhancement [B-MORE]) that was used in this study retained each of these 3 core therapeutic elements and was delivered through four 20-minute prerecorded videos. The first video provided information about pain and mindfulness before introducing an 11-minute body scan practice. The second video introduced MORE’s core mindful pain management technique. This technique helps pain patients disengage and shift attention from affective to sensory processing of pain sensations [38] and then to reorient attention to an object of their choice via mindful breathing. The third video introduced positive reappraisal and a mindful reappraisal practice exercise to familiarize participants with the mindful reappraisal worksheet. Then, participants were guided through the mindful reappraisal worksheet in the service of reappraising pain. A 5-minute mindful breathing practice was also used in this video to facilitate pain reappraisals. The fourth video introduced savoring along with a 10-minute mindful savoring practice. After each session, participants were prompted to reflect on the best parts of the mindfulness practice and any challenges that may have arisen. The final video ended with guidance for creating an at-home mindfulness practice routine.

### Data Collection

The qualitative assessment was done at the end of the 4-week intervention (6 weeks after surgery). Semistructured interviews with open-ended questions were used to encourage free expression from participants and the richness and authenticity of data [28,39]. Interviews lasted between 30 and 45 minutes and were conducted on the phone for patients’ convenience, but also to allow for data collection to take place in the participants’ natural setting as proposed by a qualitative descriptive approach [28,32]. Interviews were completed by an RA who was trained and supervised by the first author (GM), who has expertise in qualitative research and were audio recorded before being professionally transcribed. Field notes were taken by the RA as needed during interviews to allow for complementary information during the analysis. As part of the larger project, participants first rated the intervention components in terms of four attributes: (1) appropriateness in helping patients manage pain, (2) effectiveness in promoting pain self-management, (3) suitability, and (4) willingness to adhere, with the use of the treatment acceptability and preference (TAP) questionnaire [40,41]. The TAP questionnaire relies on a 5-point scale ranging from not at all (0) to very much (4). It has demonstrated adequate internal consistency (alpha >0.80) [41] and was previously validated in a surgical population receiving a web-based intervention for pain [13]. Patients’ rating of each component was used to elicit additional feedback on their perception of the intervention’s acceptability and on the need for further modifications during interviews. Table 1 presents the semistructured interview guide. These questions were not pilot tested in this study, but they were used and validated in previous acceptability studies conducted by the principal investigator (PI) [12,13,42].

**Table 1.** Semistructured interview questions based on the treatment acceptability and preference questionnaire’s attributes.

Themes	Questions
Effectiveness	What do you find the most/least helpful about the intervention? In what way do you think the intervention helped/did not help you manage your pain after surgery? In what way do you think the intervention helped/did not help you decrease the impact of pain on your recovery? In what way do you think the intervention helped/did not help you improve your ability to do your postoperative exercises?
Appropriateness	What do you find appropriate/not appropriate about the intervention? What strategies seem appropriate/inappropriate to manage postoperative pain? In what way are the strategies appropriate/not appropriate to pain management after surgery? What additional information (if any) would you like covered by the intervention?
Suitability	What pain management strategies in the intervention do you find easy to apply/not easy to apply? What do you think of the timing of the intervention? What do you think of the length of the intervention? What do you think of the therapist?
Willingness to adhere	What is easy/not easy about completing the intervention? What (if anything) could be done to make the intervention more convenient?

### Data Analysis

Descriptive statistics were used to summarize the sociodemographic and clinical characteristics of the participants at baseline and postintervention. Transcripts of the semistructured interviews were content analyzed to identify patterns and generate themes related to participants’ experiences with the intervention and their perception of its acceptability [43]. More specifically, our content analysis approach based on Miles and Huberman [43] included the following phases: coding of data, recording of insights and reflections on the data, identifying similar phrases, patterns, themes, sequences, and important features, searching for commonalities and differences, progressively deciding on generalizations that hold true for the data, and examining these generalizations in the light of existing knowledge. Our content analysis process aligned with a qualitative descriptive method by being focused on staying close to data and informants’ perspectives while also allowing the emergence of themes present across all interviews [28,29,39].

To address critiques about the subjectivity of qualitative descriptive work and enhance its rigor, steps were taken to preserve authenticity and integrity during analysis [39], and a combination of deductive and inductive approaches to coding was used. Although a particular theoretical view was not adopted to ensure data-driven coding and categorizing, a deductive approach was used to reduce researcher’s subjectivity with a preliminary generation of codes that were based on the attributes of acceptability highlighted by Sidani et al [40,41] which are directly aligned with the questionnaire used before the interviews: effectiveness, appropriateness, suitability, and willingness to adhere. Definitions of these attributes are presented in Table 2. Additional codes (subcategories) were created inductively as necessary. Reliability was ensured by performing double coding. The PI and the RA independently coded interviews.

**Table 2.** Definition of acceptability attributes (Sidani et al [40]).

Attributes	Definition
Effectiveness	Patient’s perception of the extent to which the treatment is helpful
Appropriateness	Patient’s perception of the overall treatment’s reasonableness/logicalness
Suitability	Patient’s judgment of the treatment’s intrusiveness, consistency with individual lifestyle
Willingness to adhere	Patient’s perception of the extent to which they are willing to follow the treatment

Essentially, transcripts of interviews were read several times by the PI and RA, and the content of these transcripts was first categorized under each acceptability attribute (effectiveness, appropriateness, suitability, and willingness to adhere), giving way to a more focused form of the data to help identify key themes and patterns. Then, the identification of redundant phrases and themes led to the emergence of a new category (eg, pain acceptance) before searching for differences and commonalities under each new category (eg, feeling frustrated vs feeling in control). Results were then compared, and differences were discussed until a consensus was reached. If needed, the co-PI (AH) was involved in the consensus meeting. When a new code was generated, it was discussed as well. Last, it is important to note that the researcher’s bias was also reduced by not involving the PI in the interviews. These strategies are consistent with the Consolidated Criteria for Reporting Qualitative Research (COREQ; Checklist 1) [44].

## Results

### Sample Characteristics

Participants in our qualitative sample (n=18) were older adults (mean age 67.44 years, SD 6.22 years), mostly women (13/18, 72.2%), White/Caucasian (14/18, 77.8%), and undergoing a total knee replacement (14/18, 77.8%). Table 3 shows the sociodemographic profile of the participant subset. Of note, B-MORE participants, when compared with controls, reported significantly higher baseline levels of pain interference (*d*=−0.74, *W*=93.5, *P*=.03) and anxiety (*d*=−0.61, *W*=108, *P*=.04). Out of the 18 participants completing the brief MBI intervention, 16 individuals completed the postintervention assessment. No adverse events were reported during the study. For contextual purposes, statistically significant differences were found in favor of the B-MORE participants, with lower pain intensity, pain interference, and medication use observed at follow-up. Regarding the acceptability of quantitative ratings, detailed results are presented in the article reporting the RCT [26]. Overall, participants judged B-MORE as acceptable (total mean score 3.32, SD 0.73) based on how effective, appropriate, and suitable they perceived it, as well as their willingness to adhere to it. Of note, the perceived appropriateness of the intervention received the highest score (3.5, SD 0.73), and their willingness to adhere was the lowest score B-MORE received (3.19, SD 0.75) as reflected by the TAP questionnaire.

**Table 3.** Sociodemographic characteristics of participants at baseline (n=18).

Characteristic	Value
Age (years), mean (SD)	67.44 (6.22)
Civil status, n (%)	
Divorced/separated	3 (16.7)
Married	11 (61.1)
Single	3 (16.7)
Widowed	1 (5.5)
Gender, n (%)	
Female	13 (72.2)
Male	5 (27.8)
Race, n (%)	
Native American	0 (0.0)
Black or African American	4 (22.2)
White	14 (77.8)
Living, n (%)	
Alone	4 (22.2)
With other family members	14 (77.8)
Education, n (%)	
College	7 (38.9)
Graduate School	2 (11.1)
High School	9 (50.0)
Employment, n (%)	
Full Time	0 (0.0)
On Leave	1 (5.6)
Retired	16 (88.9)
Unemployed	1 (5.6)
Surgery type, n (%)	
Total hip replacement	4 (22.2)
Total knee replacement	14 (77.8)
Currently involved in a rehabilitation program, n (%)	
No	2 (11.1)
Yes	16 (88.9)

### Acceptability Perception of B-MORE

Based on our content analytic approach, we categorized our findings related to the perception of the intervention’s acceptability into 4 main themes: perception of the effectiveness of the intervention, perception of the appropriateness of the intervention, perception of the suitability of the intervention, and perception of their willingness to adhere to the intervention. We present the categories that emerged for each theme. Table 4 presents a summary of results along with additional excerpts from interviews.

**Table 4.** Results from the content analysis on patients’ perception of the intervention’s acceptability (n=16).

Acceptability attribute and category	Verbatim (Participant ID)
Effectiveness	
Mindfulness practice	“I’d say [the most helpful was] practicing and concentrating on things other than the pain.” [P5] “When you had to stop and repeat the strategy in the middle of thinking” [P14] “I had a mental and physical feeling of warmth” [P4]
Pain acceptance	“In the beginning I was totally frustrated […] I think the questions were set up just right for the timing.” [P9] “[…] that made you aware there would be pain, but it won’t last forever.” [P1]
Supplementary relief	“[the strategies] gave me an opportunity to try another method of pain control, especially when I lay down at night” [P11] “Pain medication was the most helpful for me” [P8]
Appropriateness	
Relevance	“I found it appropriate to concentrate on things other than the pain” [P8] “I thought the strategies were fine” [P10]
Challenges	“The first one we had to look at something and focus on it. That was quite easy. After that, it seemed to get into spaces where I wasn’t sure where I was going.” [P14] “I don’t think I followed the last one correctly” [P6]
Suitability	
Timing/schedule	“I thought the timing was really good” [P10] “It’s been in a very timely and appropriate time frame” [P12]
Length	“I like the shorter ones because they are easier to go through and concentrate on” [P7] “Long enough but not so long that you couldn’t stay focused” [P10]
Technology	“It was easy to do throughout the day since I had it on my phone” [P15] [it’s] difficult […] to steady myself down” [P13]
Willingness to adhere	
Engagement	“Really, you just got to make up your mind to do the sessions and put in the effort so that it works” [P3] “You just need to make time for it” [P11]
Readiness	“I was definitely surprised to see that I was able to change the focus and that it was helpful” [P3] “I thought it would be too long and detailed” [P7] “I don’t dwell on pain” [P13]

### Effectiveness of the Brief Web-Based MBI

#### Overview

Many patients expressed positive feedback on the program’s effectiveness in helping them manage pain postsurgery. When asked about how the intervention was helpful, 3 categories were delineated in participants’ responses: mindfulness practice, pain acceptance, and supplementary relief.

#### Mindfulness Practice

Mindfulness practice was central to participants’ experiences in B-MORE, as many highlighted the usefulness of specific mindfulness techniques in managing their discomfort and improving their focus. Mindfulness practices such as body scanning (session #1), mindful breathing (session #2), and savoring (session #4) helped patients redirect their attention away from pain and toward a sense of calm and control. One participant noted, “The relaxation part where you start at the top and move down to each segment […] took my mind off the pain” [P15], illustrating how structured mindfulness practices helped shift their focus to the present moment rather than the physical sensations of pain. Additionally, participants described the value of concentrating on pleasant or neutral thoughts rather than pain, with one participant explaining, for example, that “[the most helpful part of the program was] concentrating on pleasant things rather than pain” [P9]. Another participant mentioned, “I was focusing on what I needed to do so it was relaxing” [P7]. Breathing exercises were also frequently mentioned, with some participants using mindful breathing regularly as a way to remain grounded during daily activities. For example, one participant stated that “the breathing exercises were easy to apply and think about throughout the day” [P5]. Through these mindfulness techniques, patients found a sense of relief and relaxation, which many expressed was a beneficial addition to their postoperative recovery.

#### Pain Acceptance

Acceptance also featured prominently in patients’ reflections on B-MORE, particularly as it helped them reshape their relationship with pain. Many participants expressed that B-MORE encouraged them to acknowledge pain as a natural part of their healing journey, rather than something to be resisted or feared. This shift in mindset allowed patients to feel more in control, as they recognized pain as temporary and manageable. One participant shared, “The [mindful reappraisal] questions [in session #3] reminded me that yes, I’m going to have pain […] I think what helped me was that there were a lot of questions that made you aware there would be pain, but it won’t last forever” [P1], illustrating how the program’s emphasis on acceptance through mindful reappraisal provided reassurance and resilience in the face of discomfort. This perspective was empowering for patients, who felt that viewing pain as part of recovery made it less distressing and even motivated them to engage more actively with the mindfulness exercises. Another participant described, “In the beginning I was totally frustrated and stressed and in a lot of pain. My reaction to the questions then was different than it is today. I think the questions were set up just right for the timing.” [P9]. By accepting rather than fighting their pain, the intervention helped them cope more effectively throughout the recovery process.

#### Supplementary Relief

Several participants perceived the program as an additional method of relief rather than an essential strategy. For example, one participant commented that “it was easier to do once the pain was under control” [P8], suggesting that it was not a first-choice strategy to relieve pain. Some expressed that they appreciated having access to an additional modality of pain relief and used it according to their individual needs. For example, one person commented, “[the strategies] gave me an opportunity to try another method of pain control, especially when I lay down at night” [P11]. One respondent shared that “The [mindful] pain strategies were helpful... but the pain medication was the most helpful” [P8], highlighting that mindfulness alone wasn’t sufficient in cases where pain levels were especially high. The observed variation in participant responses indicates that while B-MORE was effective for most participants, others needed a combination of mindfulness and traditional pain management strategies to achieve optimal relief.

### Appropriateness of the Brief Web-Based MBI

#### Overview

Nearly all participants found B-MORE well-suited to their recovery needs, viewing B-MORE as a constructive tool for managing postoperative pain. The perception regarding the intervention’s reasonableness/logicalness was illustrated by 2 categories: relevance and challenges.

#### Relevance

Many appreciated the direct relevance of the different mindfulness practices, in coping with their physical discomfort. One patient stated, “[…] it seemed like everything was [relevant] to me” [P1]. Another reflected, “I thought [the program] was very well done, and you just kinda had to focus and follow through” [P12], suggesting that the exercises did not require excessive effort to implement. Others echoed this sentiment, “Everything I was told to do/did was appropriate” [P11]. Additionally, mindfulness strategies resonated with some patients as suitable life skills beyond pain management. One participant stated, “Meditation is appropriate for life and pain management” [P2], showing an appreciation for the program’s relevance both within and beyond their immediate recovery period.

#### Challenges

Although the majority of participants found B-MORE thoughtfully designed and relevant to their recovery, some participants felt certain components of the program were challenging. One individual found it difficult to follow the program, “I had trouble concentrating on everything and remembering to do them most days” [P3], and another found the first session more relatable than the later sessions, “The first [session] everything was appropriate. After that, it seemed more out there and harder to figure out” [P7]. Another participant reflected on overcoming challenges by commenting, “It started out hard to do the exercises, but it got easier as I did them” [P5]. These insights suggest that while the program’s overall approach was appropriate, adjusting the pacing and sequencing of mindfulness exercises could enhance engagement and tailoring for individual needs.

### Suitability of the Brief Web-Based MBI

#### Overview

Suitability refers primarily to how the intervention was delivered. Suitability was discussed in terms of how easy the strategies were to apply or use. Several preferences regarding the timing/schedule, the exercises, and the use of technology emerged.

#### Timing and Schedule

Participants generally felt that the timing of the B-MORE intervention (ie, beginning 2 weeks after surgery) was appropriate and well-integrated into their recovery process, although opinions varied slightly based on individual recovery progress. Some participants highlighted that having this intervention during their rehabilitation program was helpful, for instance, “in the middle of rehab, so the timing was okay” [P9]. However, some participants expressed that they would have started earlier at the time of discharge by saying, “I personally would’ve been better to have started about a week earlier” [P11], while some highlighted that “the first couple of weeks were rough to focus” [P8] or reported “trouble focusing because of pain” [P9]. Last, one participant mentioned starting before surgery but was referring to postoperative pain specifically, “I think most chronic pain is before surgery, so I think this would be helpful to start this before surgery” [P1]. Regarding the schedule, a participant found that the program’s schedule was suitable because “It was in a routine and worked fine” [P5], reflecting a positive view of the weekly pacing. Many appreciated the consistent, scheduled nature of the program, as it provided a structured approach that aligned well with their recovery. One participant noted, “I thought that was great because I think anything less than that, I’d forget” [P1], underscoring how the schedule and duration of sessions supported retention and engagement. Overall, most participants found the timing and schedule to be helpful and well-suited to their needs, although a few felt minor adjustments could enhance its alignment with the early stages of postoperative recovery.

#### Length of Mindfulness Exercises

Participants expressed a range of thoughts regarding the length of the sessions and exercises, with many favoring shorter durations. One participant noted that the shorter sessions helped maintain their interest and concentration, saying, “I didn’t get bored or want to quit.” [P1]. Another echoed this sentiment, stating, “10-minute exercises were the best” [P3], while others mentioned that “anything longer” than 10‐15 minutes made it challenging to stay focused. Some found 20-minute meditation sessions acceptable but desired “less explanation” and more practice. Overall, most participants seemed to agree that shorter exercises facilitated better concentration and engagement, with a few suggesting that some sessions felt a bit long but were still satisfactory. Overall, the feedback highlights a preference for concise sessions that balance engagement and focus.

#### Technology

A number of preferences were expressed regarding the use of technology. The asynchronous nature of the mindfulness program provided participants with the flexibility to engage in pain management exercises at their convenience, fitting seamlessly into their daily routines. This flexibility allowed our surgical patients to access mindful pain management strategies when they felt most comfortable and ready, whether that was during a quiet moment at home or while on the go, “[the strategies] gave me an opportunity to try another method of pain control, especially when I lay down at night” [P11]. One participant noted, “It’s easy to do since it’s on my phone” [P12], highlighting the convenience of accessing the sessions from any device. This on-demand availability meant that participants could practice mindfulness techniques at times when they experienced heightened pain or stress, thereby maximizing the intervention’s effectiveness. The ability to revisit the exercises as needed also empowered participants to take control of their pain management. However, patients who were less accustomed to such practices found it challenging to focus. For instance, one participant commented, “I can find a quiet spot but trying to stop and focus is the hardest part” [P13], reflecting that the lack of a real-time guide or live feedback sometimes made it difficult to stay engaged. Of note, one participant mentioned that although convenient, she preferred an in-person interaction, “I know with COVID this was not possible, but I prefer a face-to-face thing” [P7]. Overall, technology was a positive aspect contributing to the personalization of B-MORE and self-management practices.

### Willingness to Adhere

#### Overview

Participants’ willingness to adhere was driven by their commitment or engagement toward the program and their readiness for a mindfulness program.

#### Engagement

Participants expressed the importance of fulfilling their commitment and putting effort into the sessions. For example, some participants stated, “Really, you just got to make up your mind to do the sessions and put in the effort so that it works” [P3], “I said I would do it, so I did it […] it forced me to sit down with it” [P5]. Remote communication with the team (eg, emailed links and reminders via text messages) and the digital relationship with the therapist also contributed to and supported that engagement. As one participant commented, “I think y’all stayed in touch” [P12]. Another one referred to the therapist by saying, “I liked to listen to him talk” [P9]. Nonetheless, adherence was challenging for patients facing high or low levels of discomfort. In such cases, intense pain could overshadow the motivation to engage in mindfulness. A patient noted, “the first couple of weeks were rough to focus […] now that I’m off the medication, my mind is clearer” [P8]. Another one stated, “I had trouble focusing because of pain” [P9], showing that factors like pain intensity and fatigue could interfere with adherence to mindfulness practices. For patients like these, added support or adaptive pacing within the program might help them maintain adherence, even during particularly challenging recovery phases. In contrast, one participant reported that their pain level had decreased to such a degree during the 4-week intervention that they no longer needed the intervention, “I think that personally my pain had decreased so I don’t know how I related to it as much” [P4].

#### Readiness

Readiness to engage in a MBI was also described by several participants. Expectations and personal beliefs were 2 aspects that were reflected when discussing readiness and adherence to the intervention. Regarding expectations, several participants expressed some uncertainty and pessimism that could have influenced their adherence initially by mentioning that they did not know what to expect and that they did not expect the intervention to be effective. They also mentioned that this perception changed throughout the intervention by commenting that what they got out of their participation was surprising and encouraging and that some strategies got easier to practice with, for instance, “It was really strange because I didn’t have a lot of expectation that it would be a big help […] just the fact of being able to focus, I found that very encouraging [P12]. Of note, one participant with previous experience with mindfulness stated, “I thought it was easy to focus because I’ve done this before […] I’m used to doing longer meditations with music […] it left me wanting more” [P2]. In this case, although readiness does not seem to be an issue and the participant would be expected to adhere easily to the intervention, having previous experience and mindfulness skills could have led to different expectations and hindered her interest in pursuing the intervention and adhering to the full program. Personal beliefs seemed to have an influence on their readiness and willingness to adhere as well. Two opposite views with 2 different impacts appeared. A personal belief that meditation or mindfulness is beneficial naturally attracted some individuals to the intervention. Nevertheless, a few participants expressed that pain was of minimal importance, which could have hindered their willingness to adhere to the intervention, “I am active and busy […] I don’t have time to dwell on pain. I get up and on with my day” [P7].

In summary, while most patients viewed the mindfulness-based program as an acceptable and valuable approach to pain management, their feedback highlights areas where additional support or customization could enhance effectiveness, appropriateness, suitability, and adherence. Tailoring session length, offering guidance for those in severe pain, and potentially providing more interactive elements could strengthen the program’s acceptability across patient experiences.

## Discussion

This is the first study to use a qualitative descriptive approach to explore the acceptability of a brief, Web-based MBI in the postdischarge recovery phase after TJA. Content analysis of the semistructured individual interviews clearly revealed that participants found the brief MBI helpful. These findings support and extend results from a recent pilot RCT, in which the brief MBI significantly reduced pain intensity, pain interference, and pain medication use relative to an active control condition [26]. Specifically, qualitative data from this study indicates that after completing the 4-week MBI, participants reported increased mindfulness leading to less concentration on the pain and more relaxation, increased pain acceptance leading to a positive attitude, and last, supplementary pain relief. Results from this study also align with previous qualitative studies on the benefits of MBIs for improved coping and relaxation among adults preparing for TJA [14] and for the management of postoperative pain and negative emotions during recovery from lumbar spine surgery [27].

Principally, results from this study contribute to understanding the acceptability of innovative ingredients addressing practical barriers and facilitating the implementation of MBI in the postdischarge period after TJA. First, the schedule of 4 weekly sessions starting 2 weeks after surgery seems to fit the recovery process and needs of most participants while promoting their participation as the worst pain levels are usually experienced within the first 2 weeks after surgery [45], and 6 weeks is the timeframe for normal tissue healing and recovery [46,47]. A few participants mentioned the possibility of starting earlier, as the pain did not seem to hinder their participation or had subsided quickly after starting the program. This aligns with a previous study with orthopedic postsurgical patients in which participants expressed this preference as they would have used the MBI strategies for other aspects of recovery than pain [27]. Second, the brief format (20 min sessions for 4 weeks), including short meditations, seemed to be appreciated by participants, which responds to feasibility and acceptability issues encountered in previous studies implementing a traditional 8-week format before surgery in this population [14,15]. Nevertheless, the only study assessing acceptability in the recovery phase after orthopedic surgery (lumbar spine) reported that 75% of patients were satisfied with 8 weekly sessions of over an hour [27]. Third, remote delivery in the postoperative period seems to offer more than convenience for participants with limited mobility; it makes their participation possible after a major surgery [12,27,48]. However, our program also addressed time and logistical constraints met by a variety of individuals [15,49-52]. As expressed by some participants, in addition to remote delivery, the asynchronous nature of the intervention allowed for even more convenience with accessibility to the program anytime from anywhere, according to individual needs and pain levels during recovery. Indeed, a previous study with adults after lumbar spine surgery reported that 25% of their participants had trouble finding an uninterrupted space at the time their session was scheduled [27]. Offering continuous access to therapeutic content acknowledges these differences during recovery [53]. Last, reminders also contributed to the acceptability of our program. As highlighted by others who evaluated the acceptability of Web-based MBIs qualitatively, check-ins are desired by most participants in order to maintain adherence and promote home practice [54] and call for hybrid approaches [12,55-57].

While participants identified multiple benefits and found the brief MBI appropriate during the early recovery period, they also noted some challenges that impacted their adherence. The main challenges were an initial uncertainty about what to expect from a “mindfulness” intervention and skepticism about its effectiveness. This hesitancy stemmed from mindfulness being unfamiliar to most participants, including reliance on traditional pharmacological approaches. This finding is resonant with the quantitative phase of this study, where the willingness to adhere item received the lowest rating on a multidimensional, Likert-type acceptability survey, despite no participant reporting unwillingness. This finding also converges with a recent qualitative analysis in which the impact of preoperative mindfulness-based stress reduction for patients with TJA was linked to their “openness” toward an MBI, health-related beliefs, and their motivations for participation [14], as well as another qualitative evaluation of an 8-week MBI after lumbar spine surgery in which some participants reported that the purpose was unclear and that they would have benefited more from the program had they understood earlier [27]. Other studies, both qualitative and quantitative, have also identified similar challenges, with participants reporting difficulty understanding the purpose of mindfulness practice [58], doubting the value or benefits [59], or dropping out of MBI groups at a higher rate than control groups, including some participants not attending any sessions [60,61]. Thus, by proactively addressing expectations and beliefs about mindfulness’s ability to manage pain, we can reduce skepticism, foster greater acceptance, and enhance participant engagement and adherence from the outset. Of note, the MORE approach [34], used as a foundation for the brief intervention in this study, uses principles of reappraisal (cognitive-behavioral therapy) and savoring (positive psychology) to disrupt the spiral of chronic pain characterized by maladaptive thoughts and behaviors, including overreliance on pain medication. MORE targets populations facing opioid misuse [35,37].

Furthermore, while addressing initial expectations and beliefs is crucial for increasing readiness and reducing uncertainty about any treatment, a few participants identified known challenges unique to mindfulness practice [62]. Indeed, qualitative studies involving participants with various backgrounds and varying levels of familiarity with mindfulness have described challenges related to mindfulness practice [50,51,63]. It is not unusual for individuals to doubt their skills and knowledge. In this study, several participants reported having difficulty focusing, following some sessions, or feeling unsure of “spaces” they were “getting into.” Mind wandering and feeling strange can be perceived as a barrier to mindfulness practice [50-52,59] and could impede participants’ adherence to the intervention. Connecting with negative feelings is another barrier, as they can be challenging or overwhelming at times [49,51,63]. In this study, some participants reported similar barriers, expressing difficulty practicing mindfulness while experiencing elevated pain or during moments of general discomfort or frustration. Proactively providing participants with information about common challenges during mindfulness practices, as well as techniques for mitigating those challenges, would likely further enhance the acceptability and effectiveness of the brief MBI.

This study has several limitations. First, the homogenous sample limits our understanding of perceptions across diverse sociodemographic groups (older adults, White, female). Additionally, the reported perceptions are from individuals who agreed to participate, and 2 participants dropped out of the experimental group. Although a multilevel meta-analysis of RCTs using the MORE program for other conditions highlighted that diverse groups, including younger adults (mean age 25‐59 years) as well as underrepresented racial/ethnic groups, have received this intervention [64], future qualitative studies could use quota sampling to include the perception of various subgroups, including those who refused to participate, thus enhancing our understanding of barriers to the intervention’s uptake. While not necessarily a limitation, participants noted that postoperative pain medication might have hindered their ability to follow the sessions, and that mindfulness practice was more challenging during pain spikes. Last, the qualitative analysis did not account for the time participants spent watching the videos or practicing mindfulness. Consequently, the level of comfort with the intervention may have been influenced by the amount of practice. Comparing acceptability attributes based on the amount of practice could provide valuable insights.

However, several barriers to adherence emerged in relation to the challenges of mindfulness practice in the postoperative recovery period, and these warrant further exploration to ensure the brief MBI’s success in a future, fully powered, RCT. For instance, it would be interesting to assess if additional digital health tools, such as chatbots, would be beneficial in offering a more tailored support for patients who find mindfulness practice challenging during pain spikes. Furthermore, regarding the timing of the intervention, although we targeted a specific subgroup based on risk factors for CPSP (ie, pain<4/10 at 2 wk), some participants felt ready to start earlier, and some would have started later during recovery. Future studies could explore additional variables to help determine who would benefit from which timing of intervention or compare the value of different timings of intervention during postoperative recovery. Moreover, although the brief format (ie, 4-week sessions of 30 min) represented a positive aspect of the intervention, would some postoperative patients benefit from a full standard format of 8 weekly sessions of one hour?

Given the increasing number of TJAs performed each year and the ability of nonpharmacological interventions, such as MBIs, to support recovery and well-being, efforts should be made to increase patient access to these promising adjunctive treatments. In this study, semistructured interviews indicated that the brief MBI promoted self-management of pain during the early recovery period. Additionally, while most studies to date have tested the implementation of brief preoperative interventions for patients with TJA when postoperative pain is not present yet, our study highlights the acceptability of a postoperative MBI. Combining MBI interventions before and after surgery at different key time points of the perioperative continuum of care may be an interesting avenue to optimize pain relief and recovery, as well as prevent complications such as the development of CPSP. Finally, the use of technology could improve the accessibility, scalability, and adoption of these promising approaches.

## Supplementary material

10.2196/69010 Checklist 1 COREQ (Consolidated Criteria for Reporting Qualitative Research) checklist.

## Abbreviations

B-MORE: brief mindfulness-oriented recovery enhancement
COREQ: Consolidated Criteria for Reporting Qualitative Research
CPSP: chronic postsurgical pain
MBI: mindfulness-based intervention
MORE: mindfulness-oriented recovery enhancement
PI: principal investigator
RA: research assistant
RCT: randomized controlled trial
TAP: treatment acceptability and preference
TJA: total joint arthroplasty
